# Reproductive inhibition among nestmate queens in the invasive Argentine ant

**DOI:** 10.1038/s41598-020-77574-1

**Published:** 2020-11-24

**Authors:** Sílvia Abril, Crisanto Gómez

**Affiliations:** Department of Environmental Sciences, University of Girona, M. Aurèlia Campmany, 69, 17003 Girona, Spain

**Keywords:** Entomology, Invasive species

## Abstract

In social species, the presence of several reproductive individuals can generate conflict. In social insects, as queen number increases, individual oviposition rate may decrease because of direct and indirect behavioural and/or chemical interactions. Understanding the factors that mediate differences in queen fecundity should provide insight into the regulation and maintenance of highly polygynous insect societies, such as those of the invasive Argentine ant (*Linepithema humile*). In this study, we investigated (1) whether differences in the oviposition rates of Argentine ant queens exposed to polygynous conditions could result from interactions among them; (2) whether such differences in fecundity stemmed from differences in worker attention; and (3) whether polygynous conditions affected the cuticular hydrocarbon profiles of queens (CHCs). We found that differences in queen fecundity and CHC profiles observed under polygynous conditions disappeared when queens were exposed to monogynous conditions, suggesting some form of reproductive inhibition may exist when queens cohabit. These differences did not seem to arise from variation in worker attention because more fecund queens were not more attractive to workers. Levels of some CHCs were higher in more fecund queens. These CHCs are associated with greater queen productivity and survival. Our findings indicate that such compounds could be multifunctional queen pheromones.

## Introduction

Reproductive division of labour is a key feature of insect societies. It is a phenomenon in which only a small number of colony members reproduce; other colony members rear the offspring, to whom they are related. However, the presence of several reproductive individuals within colonies (queens and/or workers) generates conflict over reproduction^1^. For example, in bumble bees^2^, honey bees^3,4^ and wasps^5^, the presence of a queen can inhibit egg-laying by workers. In termites^6,7^, it can also decrease egg production by queens and suppress the differentiation of new female neotenics, and, in ants, it can inhibit the reproduction of workers and gynes (winged virgin queens), reduce the fertility of nestmate queens, and regulate diploid brood sexualisation^8^.

In polygynous ant societies, as a result of queen-queen competition, an increase in the number of queens^9,10^ can lead to a decrease in queen fecundity. There are two likely mechanisms by which higher queen number could diminish individual oviposition rate: (1) exploitative competition—as queen number increases, each queen may receive less food, limiting her egg production and (2) direct and indirect behavioural and/or chemical interactions^8^.

In the highly polygynous Argentine ant, *Linepithema humile* (Mayr, 1868), it has been observed that queen fecundity is inversely proportional to the number of queens in the colony^11–13^. In addition, under experimentally induced conditions of polygyny, queens were found to vary in their oviposition rates: one or two queens laid almost all the eggs for the colony, while the other queens contributed few to no eggs^13,14^. There is also evidence that the presence of mature queens strongly inhibits the production of nestmates. For example, Passera et al.^15^ found that the production of males is affected by queens via food appropriation. Vargo and Passera^16,17^ discovered that mature queens strongly decreased the production of gynes in two ways: (1) by preventing the sexualisation of female larvae by exerting a pheromonal influence over the brood-rearing behaviour of workers and (2) by killing female larvae after they had become sexualised. Execution of larvae appeared to be mediated, on one hand, by a primer pheromone that elicited execution behaviour from workers and, on the other hand, by the queen directly executing gyne larvae herself. Mated queens can also inhibit egg-laying by virgin queens by preventing virgin queen dealation^18^. These results underscore that some form of reproductive inhibition may exist among Argentine ant queens that influences individual oviposition rates under polygynous conditions. That said, when Keller^11^ compared the oviposition rates of queens exposed to monogynous versus polygynous conditions, he found that highly fecund queens were equally common in both situations. These results suggested that there were no dominance hierarchies among Argentine ant queens and that the marked variability in queen fecundity seen under polygynous conditions arose from intrinsic physiological differences among queens. However, given that Keller did not experimentally manipulate the queens’ conditions, it was impossible to determine (1) whether the differences in an individual queen’s fecundity disappeared when the queens were alone and (2) whether behavioural or physiological variability could result from interactions between individuals.

Consequently, in this study, we wished to determine whether there was evidence that differences in the oviposition rates of Argentine ant queens exposed to polygynous conditions could result from interactions among individuals, which would suggest that queens engage in a form of reproductive competition. We explored this question experimentally: we exposed queens to both polygynous and monogynous conditions and examined their oviposition rates and ovarian activity. We hypothesised that if differences in fecundity among queens stemmed from intrinsic physiological differences, queens would display the same levels of fecundity under both types of conditions.

Several studies in various species have found a correlation between queen fecundity and worker attention as well as between queen fecundity and/or the nutritional care provided to queens by workers, suggesting that workers favour more productive queens^19–21^. Keller^11^ has hypothesised that Argentine ant queens may receive less food under polygynous versus monogynous conditions, which could partially explain previously observed differences in fecundity. Here, we used behavioural observations to assess whether differences in fecundity among Argentine ant queens exposed to experimentally induced polygynous conditions arose from differences in worker attention. If queens have higher oviposition rates and are thus more fecund because they receive more food, we hypothesised that worker attention would be positively correlated with oviposition rates and ovarian index values.

Differences in the reproductive contributions of Argentine ant queens under polygynous conditions may be affected by chemical interactions, possibly involving pheromones. Indeed, in various social insect species, pheromones released by queens can regulate the reproductive rates of other queens. For example, in the termite *Reticulitermes speratus*, a queen pheromone regulated the egg production of queens, causing a significant decrease in oviposition rates^7^. In ants, in *Solenopsis invicta*, queen pheromones inhibited the fecundity of functional queens under polygynous conditions, a phenomenon that might mediate reproductive competition among mature queens^22^. Likewise, in *Lasius niger*, a cuticular hydrocarbon (CHC) identified as a multifunctional pheromone also inhibited queen fecundity^23^. The presence of this CHC was correlated with queen productivity, maturity, and survival^24^ and was an honest signal of queen fertility. In the Argentine ant, recent studies have shown similar results: the presence of specific CHCs in queens was correlated with queen fecundity and survival^25,26^, which means these compounds could also act as multifunctional queen pheromones. In this study, we characterised the CHCs of high- and low-fecundity queens under polygynous and monogynous conditions to determine whether queen CHC profiles differed.

To date, few studies have focused on the interactions among queens in polygynous colonies, and the influence of competition on the egg-laying rates of mature queens has rarely been observed^27^. Indeed, there has only been a single report of reproductive inhibition among queens in *S. invicta*^22^; queen pheromones were hypothesised to be the underlying mechanism. Furthermore, it remains unclear why queens in polygynous colonies would refrain from laying eggs in the presence of nestmate queens^8^.

To our knowledge, this is the first study to experimentally explore the proximate mechanisms of reproductive differences among Argentine ant queens under polygynous versus monogynous conditions using physiological, behavioural, and chemical data. Our study also sheds light on the factors that mediate reproduction in polygynous ant societies.

## Results

### Did nestmate queens affect the reproductive contributions of individual queens in the Argentine ant?

**Figure 1:**
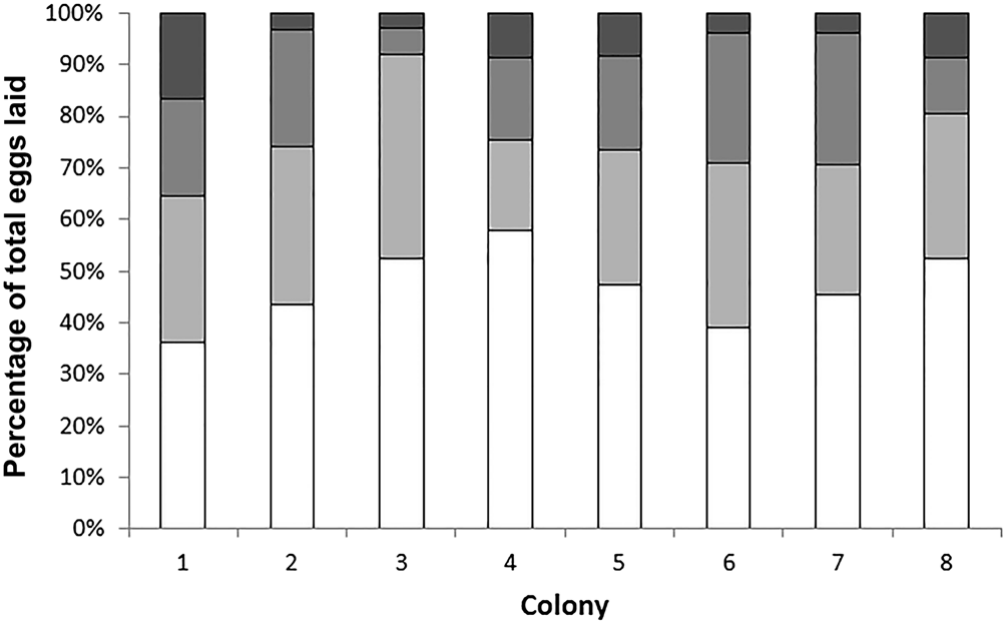
The relative reproductive contributions of the queens (i.e., % of total eggs laid) (n = 32) within each polygynous colony (n = 8). Each queen is represented by a different colour.

If egg production were to be equally carried out by a colony’s queens, we would expect each queen to lay approximately 25% of the colony’s eggs (given there were four queens per polygynous colony). However, we observed that, in almost all colonies, two queens always contributed more than 25% of the eggs. In contrast, the other two queens barely contributed any eggs (Fig. 1).

**Figure 2:**
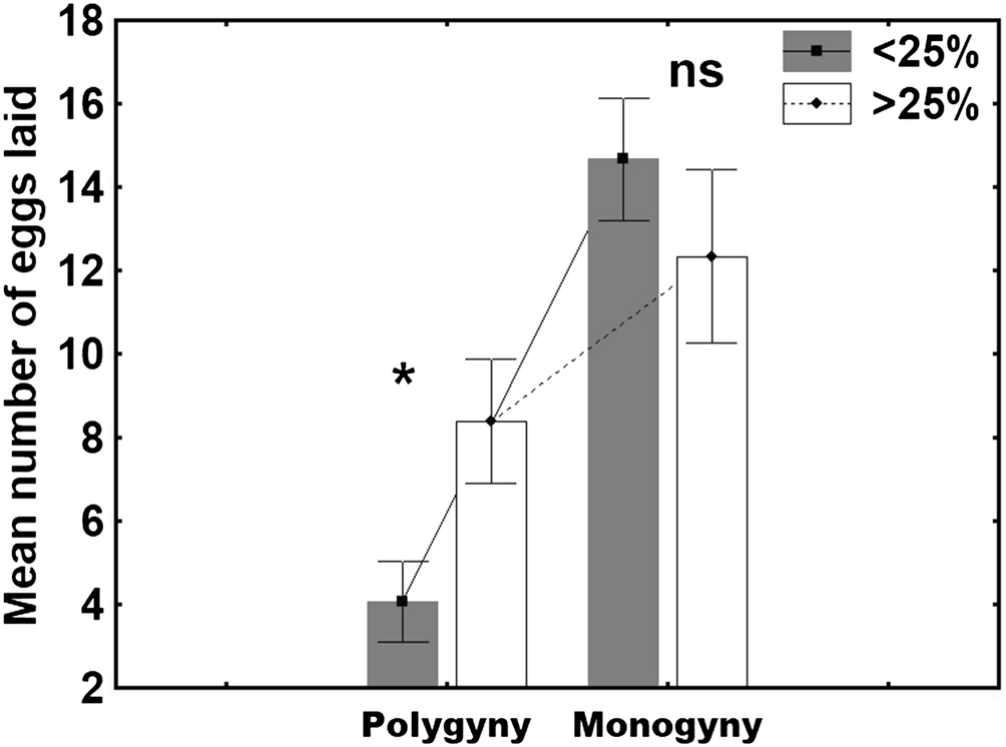
Mean number of eggs laid over a 24-h period (mean ± SE) by high-fecundity queens (> 25%; white bars) (n = 7) and low-fecundity queens (< 25%; grey bars) (n = 7) under polygynous and monogynous conditions. The asterisk denotes significant differences (*p < 0.05); ns: no significant differences.

Reproductive skew was significant in all the polygynous colonies (i.e., B-values were positive; Table 1), indicating that, effectively, certain queens contributed more to egg laying. These high-fecundity queens have higher oviposition rates than low-fecundity queens under polygynous conditions (GLMM t_48_ = 2.41; *p* < 0.05; Cohen’s *d* = 0.65) (Fig. 2)). However, the same queens did not differ in their mean oviposition rates under monogynous conditions (GLMM t_34_ = − 1.03; *p* = 0.31; Cohen’s *d* = 0.028) (Fig. 2).

**Table 1:** Estimates of reproductive skew (B-index values) among queens in polygynous colonies of the Argentine ant.

Colony	B_adj_	B-index	*p*	Minimum	Maximum
1	0.0189	0.0192	** < 0.005**	− 0.0052	0.7448
2	0.1027	0.0808	** < 0.001**	− 0.0047	0.7453
3	0.2321	0.1781	** < 0.001**	− 0.0074	0.7426
4	0.1688	0.1354	** < 0.001**	− 0.0132	0.7368
5	0.0842	0.0718	** < 0.001**	− 0.0104	0.7396
6	0.0729	0.0611	** < 0.001**	− 0.0075	0.7425
7	0.0897	0.0755	** < 0.001**	− 0.01	0.74
8	0.1476	0.1159	** < 0.001**	− 0.0073	0.7427

Positive values indicate skewed reproduction, negative values indicate shared reproduction, and values equal to zero indicate random reproductive skew. In bold are the results that differed significantly from zero. Minimum: value indicating that egg-laying is completely equal; maximum: value indicating that egg-laying is monopolized by one queen.

Egg production varied across the four trials involving polygynous conditions: almost none of the low-fecundity queens laid eggs after having resided with the high-fecundity queens for two weeks (mean egg number/24 h ± SD—trial 1: 3.16 ± 3.98, trial 2: 4.88 ± 5.15, trial 3: 3.77 ± 6.10, trial 4: **0.77 ± 1.69**). These results therefore suggest that it took approximately two weeks for the high-fecundity queens to reduce the oviposition rates of the low-fecundity queens.

The number of queens within a colony should affect the queens’ individual oviposition rates (Abril et al.^13^), such that the presence of fewer queens should result in higher individual oviposition rates. Consequently, we expected to see queen oviposition rates increase as queens went from polygynous to monogynous conditions. However, the oviposition rates of high- versus low-fecundity queens did not increase in the same way as the ants went from polygynous to monogynous conditions. High-fecundity queens displayed a 1.5-fold increase in their oviposition rates, while low-fecundity queens increased theirs 3.5 fold (Fig. 2).

**Figure 3:**
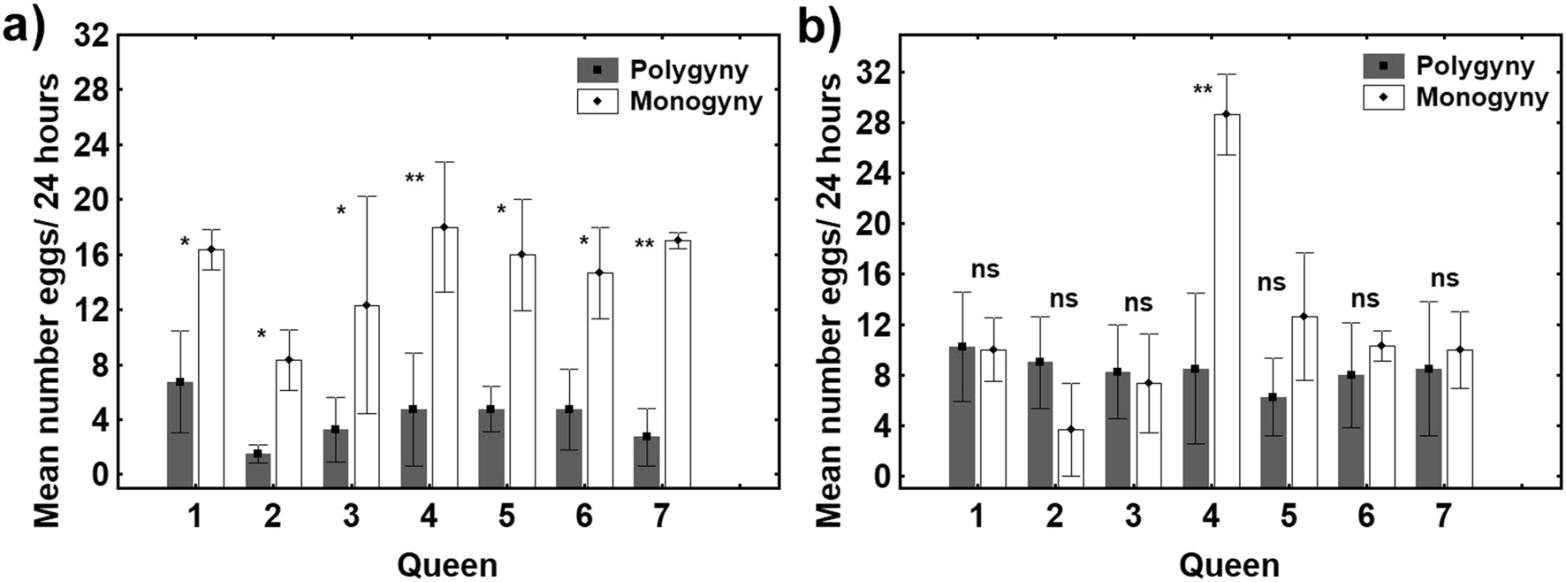
Mean number of eggs laid over a 24-h period (mean ± SE) by each low-fecundity queen (n = 7) (**a**) and each high-fecundity queen (n = 7) (**b**) under polygynous and monogynous conditions. The asterisks denote significant differences (*p < 0.05; ** p < 0.01); ns: no significant differences.

When we examined these results at the individual level, we found that all the low-fecundity queens significantly increased their oviposition rates, while almost all the high-fecundity queens simply maintained theirs (Fig. 3a,b).

**Figure 4:**
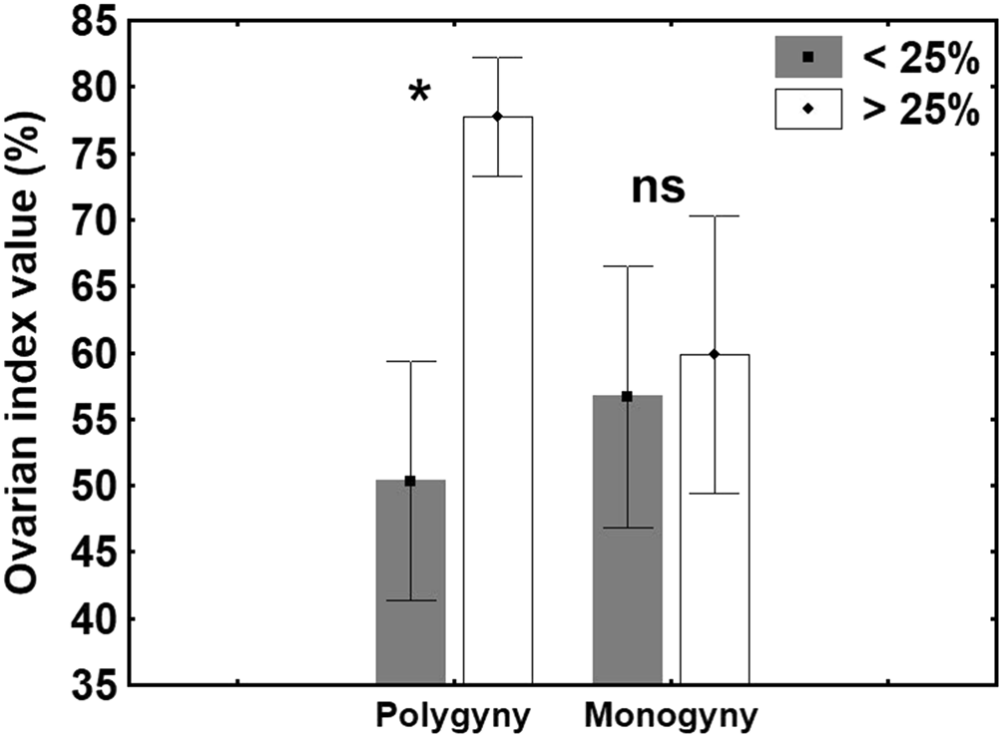
Ovarian index values (mean ± SE) of high-fecundity queens (> 25%; white bars) (n = 7) and low-fecundity queens (< 25%; grey bars) (n = 7) under polygynous and monogynous conditions. The asterisk denotes significant differences (*p < 0.05); ns: no significant differences.

As for ovarian activity, the mean ovarian index (OI) value of the high-fecundity queens was significantly higher than that of the low-fecundity queens (GLMM t_6_ = − 2.71; *p* < 0.05; Cohen’s *d* = 1.451) (Fig. 3) under polygynous conditions; no such difference existed under monogynous conditions (GLMM t_6_ = -0.218; *p* = 0.831; Cohen’s *d* = 0.116) (Fig. 4).

### Did workers pay more attention to high-fecundity queens than low-fecundity queens?

There were no correlation between queen fecundity and worker attention (GLMM t_6_ = − 1.87; *p* = 0.11; Cohen’s *d* = 0.278).

### Did high- and low-fecundity queens have different cuticular hydrocarbon profiles under polygynous versus monogynous conditions?

**Figure 5:**
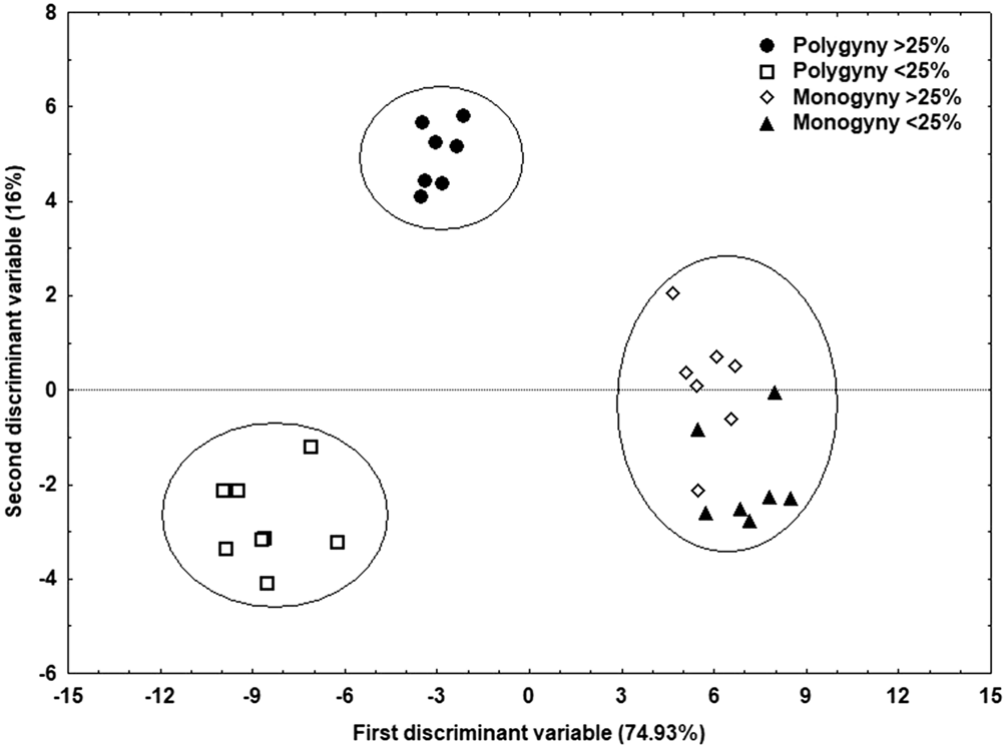
Results of the discriminant analysis conducted on the relative quantities of CHCs in the profiles of high-fecundity queens (> 25%) (n = 7) and low-fecundity queens (< 25%) (n = 8) under polygynous and monogynous conditions.

There were significant differences between the two groups under polygynous conditions (Fig. 5). This visual distinction was supported by the results of the ANOSIM analysis and the subsequent pairwise tests (CHC differences—under polygynous conditions: R: 0.15, *p* < 0.05; under monogynous conditions: R = − 0.088, *p* = 0.7916).

**Figure 6:**
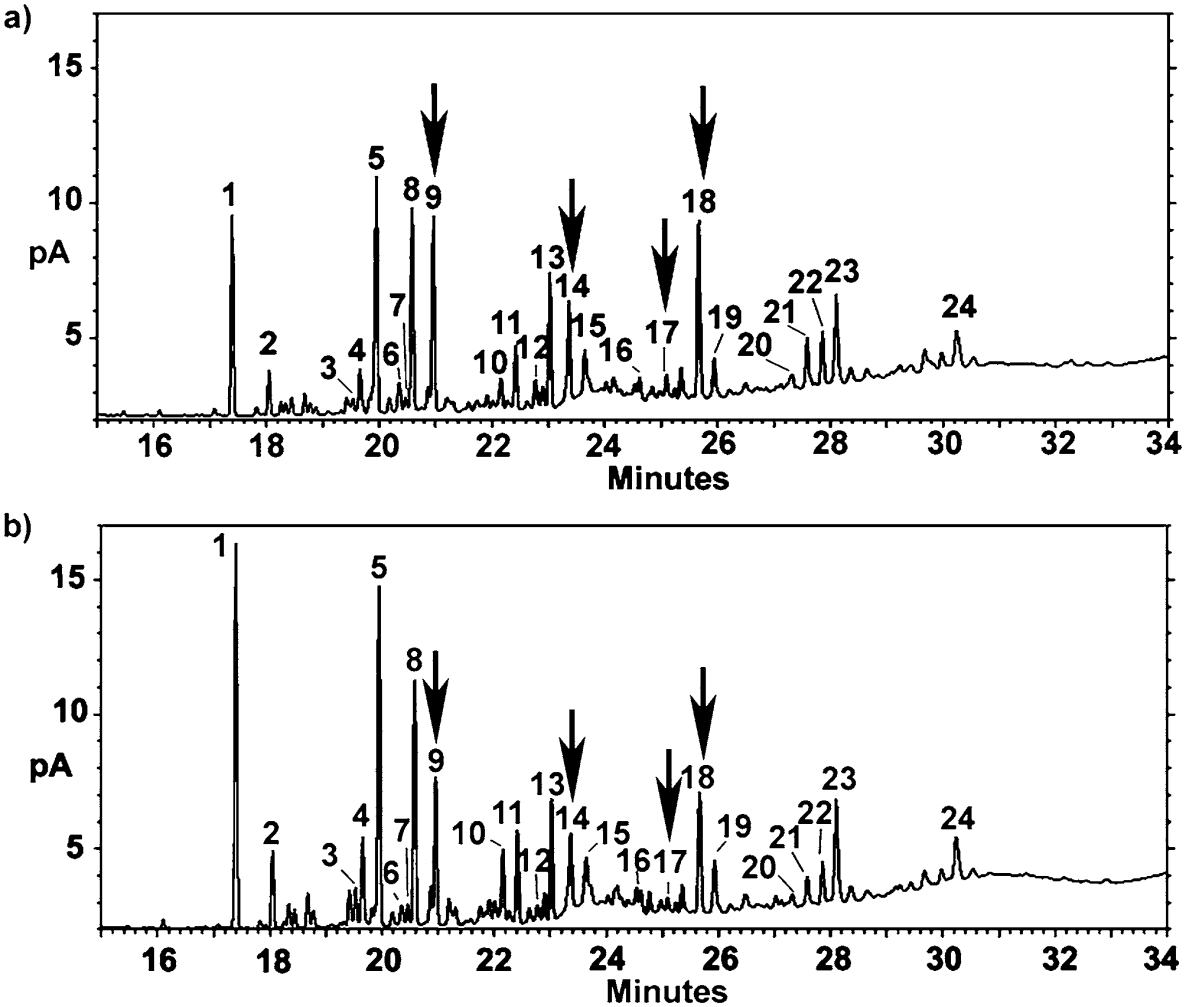
Examples of CHC profiles for a high-fecundity queen (**a**) and a low-fecundity queen (**b**). Compound names are provided in Table 2. The arrows indicate the compounds 5,11-diMeC_29_; 5, 11-diMeC_31_; 11-MeC_33_; and 5,11-diMeC_33_.

Two discriminant functions explained most of the variance (first function: R = 0.99, Wilk’s λ = 0.00025, χ^2^_69_ = 120.23, *p* < 0.005; second function: R = 0.955, Wilk’s λ = 0.0125, χ^2^_44_ = 63.48, *p* < 0.05; 96.5% correct classification). The main CHCs responsible for these differences were three di-methyl alkanes (5,11-diMeC_29_, 5,11-diMeC_31_, and 5,11-diMeC_33_), a linear alkane (C_29_), and one mono-methyl alkane (11-MeC_33_) (Fig. 6).

**Figure 7:**
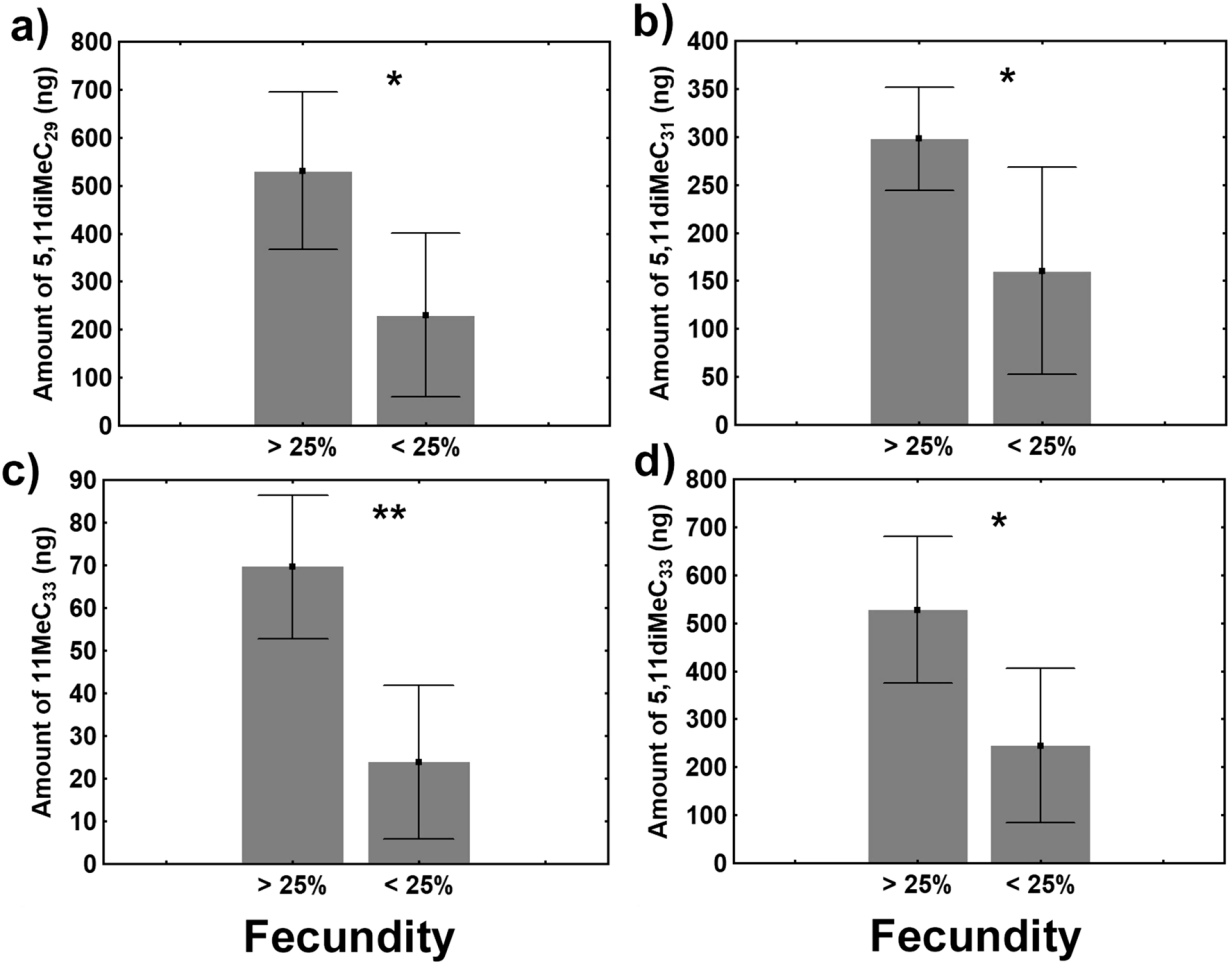
Amounts of 5,11-diMeC_29_ (**a**), 5,11-diMeC_31_ (**b**), 11-MeC_33_ (**c**), and 5,11-diMeC_33_ **(d)** in the CHC profiles of high-fecundity queens (> 25%) (n = 7) and low-fecundity queens (< 25%) (n = 8) (mean ± SE). The asterisks denote significant differences (*p < 0.05; **p < 0.01).

We therefore examined the absolute quantities of these compounds. Overall, high-fecundity queens had greater amounts of 5,11-diMeC_29_, 5,11-diMeC_31_, 5,11-diMeC_33_, and 11-MeC_33,_ than did low fecundity queens (5,11-diMeC_29_: GLMM t_6_ = -3.36, *p* < 0.05, Cohen’s *d* = 1.797; 5,11-diMeC_31_: GLMM t_6_ = − 2.98, *p* < 0.05, Cohen’s *d* = 0.355; 5,11-diMeC_33_ GLMM: t_6_ = − 3.43, *p* < 0.05, Cohen’s *d* = 1.803; 11-MeC_33_: GLMM t_6_ = − 4.92, *p* < 0.01, Cohen’s *d* = 2.630) (Fig. 7; Table 2).

**Table 2:** Relative percentages (mean ± SE) of the major cuticular hydrocarbons of high- and low-fecundity queens under polygynous and monogynous conditions.

Peak number^a^	Compound	High-fecundity queens, polygyny (n = 7)	Low-fecundity queens, polygyny (n = 8)	High-fecundity queens, monogyny (n = 7)	Low-fecundity queens, monogyny (n = 7)
1	*n*-C_27_	6.22 ± 0.43	11.40 ± 3.18	9.54 ± 1.31	7.79 ± 0.98
2	5-MeC_27_	1.77 ± 0.36	1.60 ± 0.29	1.94 ± 0.18	1.40 ± 0.14
3	xC_29:1a_	0.98 ± 0.22	0.72 ± 0.22	1.46 ± 0.16	1.09 ± 0.14
4	xC_29:1b_	2.74 ± 0.41	3.25 ± 0.77	4.32 ± 0.57	4.02 ± 0.54
5	*n*-C_29_	8.60 ± 0.67	11.57 ± 1.42	10.32 ± 0.76	10.53 ± 0.80
6	11-MeC_29_	1.52 ± 0.15	1.20 ± 0.16	1.88 ± 0.20	1.78 ± 0.13
7	7-MeC_29_	1.14 ± 0.24	1.06 ± 0.15	0.96 ± 0.50	0.94 ± 0.07
8	5-MeC_29_	7.46 ± 0.47	6.74 ± 0.76	8.07 ± 0.76	7.60 ± 0.84
9	**5,11-diMeC**_**29**_	**8.07 ± 0.61**	**5.33 ± 1.06**	8.22 ± 0.83	8.80 ± 0.84
10	xC_31:1_	1.18 ± 0.06	0.91 ± 0.16	0.71 ± 0.13	0.84 ± 0.08
11	*n*-C_31_	2.57 ± 0.23	2.67 ± 0.50	2.75 ± 0.28	2.66 ± 0.21
12	11 + 13-MeC_31_	1.31 ± 0.42	0.94 ± 0.15	0.84 ± 0.14	0.79 ± 0.16
13	5-MeC_31_	4.95 ± 0.47	3.84 ± 0.35	4.64 ± 0.48	4.51 ± 0.58
14	**5,11-diMeC**_**31**_	**4.59 ± 0.13**	**3.61 ± 0.57**	4.07 ± 0.24	4.16 ± 0.33
15	*n*-C_32_	2.56 ± 0.23	2.84 ± 0.57	1.92 ± 0.12	2.08 ± 0.18
16	xC_33:1b_	1.19 ± 0.10	0.73 ± 0.22	0.86 ± 0.18	0.73 ± 0.07
17	**11-MeC**_**33**_	**1.06 ± 0.05**	**0.57 ± 0.12**	0.91 ± 0.18	0.80 ± 0.18
18	**5,11-diMeC**_**33**_	**8.08 ± 0.65**	**5.42 ± 0.84**	6.31 ± 0.67	6.22 ± 0.57
19	*n*-C_34_	2.47 ± 0.23	2.86 ± 0.66	2.02 ± 0.09	2.26 ± 0.15
20	11-MeC_35_	1.10 ± 0.07	0.83 ± 0.15	1.15 ± 0.13	1.08 ± 0.13
21	5,11-diMeC_35a_	2.13 ± 0.12	1.65 ± 0.22	2.28 ± 0.12	2.42 ± 0.18
22	5,11diMeC_35b_	2.51 ± 0.20	1.87 ± 0.27	2.13 ± 0.22	2.20 ± 0.23
23	5,13,17 + 5,15,19-triMeC_35_	4.76 ± 0.47	5.46 ± 1.16	3.31 ± 0.25	4.13 ± 0.36
24	x,y,z-triMeC_37_	3.43 ± 0.48	2.44 ± 0.60	1.57 ± 0.29	2.29 ± 0.24

Statistically significant results are in bold.

^a^Compounds are ordered by retention time. See the peaks associated with the numbers in Fig. 6.

We also looked at the correlations between the absolute quantities of these four compounds and both oviposition rates and OI values. We found that the amounts of all four hydrocarbons were positively correlated with the two variables (oviposition rate—5,11-diMeC_29_: Pearson’s r = 0.714, *p* < 0.0001; 5,11-diMeC_31_: Pearson’s r = 0.625, *p* < 0.0001; 5,11-diMeC_33_: Pearson’s r = 0.640, *p* < 0.0001; and 11-MeC_33_: Pearson’s r = 0.703, *p* < 0.00001; OI—5,11-diMeC_29_: Pearson’s r = 0.477, *p* < 0.05; 5,11-diMeC_31_: Pearson’s r = 0.621, *p* < 0.0001; 5,11-diMeC_33_: Pearson’s r = 0.576, *p* < 0.01; and 11-MeC_33_: Pearson’s r = 0.515, *p* < 0.01).

In contrast, under monogynous conditions, high- and low-fecundity queens did not differ in their CHC profiles—the two groups clustered together in the discriminant analysis (Fig. 5).

## Discussion

Our results indicate that some form of reproductive inhibition exists among nestmate queens in the Argentine ant. First, under polygynous conditions, there was variability in oviposition rate and ovarian activity: we saw distinct groups of high- and low-fecundity queens. These results concur with those of previous studies, which also found differences in the oviposition rates of Argentine ant queens under polygynous conditions^13,14^. In contrast, under monogynous conditions, these differences in fecundity disappeared. Second, if there were no reproductive inhibition among queens, we would have expected to see a proportional increase in oviposition rates for all queens under monogynous conditions, since a negative relationship has been found between queen number and oviposition rate^9,10^. Instead, we observed that the oviposition rates of low-fecundity queens increased 3.5 fold, while the oviposition rates of high-fecundity queens increased just 1.5 fold. This finding suggests that queen fecundity was inhibited under polygynous conditions. There is some evidence that Argentine ant queens pheromonally inhibit the production of gynes and the dealation of virgin queens^16–18^. Therefore, it is possible that a queen-produced pheromone could mediate the differences we observed in queen fecundity under polygynous conditions. This hypothesis is based on the fact that queens in other ant species use queen pheromones to directly inhibit each other’s reproduction. For example, in *Solenopsis invicta*, a primer pheromone was found to inhibit the dealation of virgin queens^28^ and to inhibit the fecundity of functionally reproductive queens^22^. In *Lasius niger*, mature queens may use queen pheromones to reduce their own reproductive output when other queens and their brood are present^23^.

It has been hypothesised that reproductive competition among queens is mediated by a pheromone that operates directly on queen physiology^22^. In this study, low-fecundity queens had lower OI values than did high-fecundity queens under polygynous conditions but not under monogynous conditions. Therefore, it is possible that fecundity-reducing pheromones could act directly on Argentine ant queen physiology by reducing their ovarian activity, suggesting some form of reproductive competition among queens in this species. This hypothesis is supported by the fact that Argentine ant queens have been found to have a low degree of relatedness^29,30^, perhaps in part because they change nests frequently^31^. Therefore, by inhibiting the reproductive activity of their rivals, high-fecundity queens may be able to increase their personal fitness by securing a greater proportion of the colony’s resources to rear their own progeny. However, Keller and Nonacs^32^ have suggested that such a mechanism is unlikely because a pheromone that directly suppressed egg production would not spare the queen emitting it. Vargo^22^ has asserted that such a system remains possible as long as (1) the amount of pheromone released is positively correlated with queen fecundity and (2) sensitivity to the pheromone’s effects is negatively correlated with fecundity. In other words, more fecund queens would contribute more to the amount of the inhibitory pheromone but would be less affected. *Lasius niger* appears to use a system of this type, in which a queen pheromone that inhibits worker reproduction also negatively affects queen productivity^23^. This pheromone is a cuticular hydrocarbon (3-MeC_31_) and meets the criteria established by Vargo^22^: the amount of this compound is positively correlated with queen fecundity^33^, and sensitivity to its effects is negatively correlated with fecundity because workers are strongly affected^24^, but queens are weakly affected^23^. Recently, it was shown that several queen CHCs, including the di-methyl alkanes 5,11-diMeC_29_, 5,11-diMeC_31_ and 5,11-diMeC_33_ were positively correlated with queen fecundity^25^ and negatively correlated with queen executions^26^ in the Argentine ant. In this study, high- and low-fecundity queens had significantly different levels of 5,11-diMeC_29,_ 5,11-diMeC_31_, and 5,11-diMeC_33_ under polygynous conditions, indicating that these compounds may serve as multifunctional pheromones in the Argentine ant.

Further research is necessary to assess the effects of these compounds on queen physiology and worker behaviour to see if they are actually queen pheromones or if they simply signal queen fertility. It could also shed light on the compounds’ mode of action.

Keller and Nonacs^32^ have asserted that direct pheromonal inhibition of oviposition among queens should be evolutionarily unstable given that there would be strong selection on target individuals to avoid inhibition. Therefore, it has been hypothesised that such a pheromone should act indirectly, by causing workers to feed queens less food and/or other substances required for egg production^22^. It could operate by changing queen attractiveness and thus modifying the number of workers attending to a given queen. As a consequence, dominant queens might attract more workers, receive more food, and produce more eggs than subordinate queens, a phenomenon that has already been observed in some ant species^19,20,34^. However, in this study on the Argentine ant, more fecund queens were not more attractive to workers.

It is therefore possible that, in this particular species, the differences in fecundity among queens under polygynous conditions are driven by direct pheromonal inhibition of queen physiology. A queen’s real physiological state might thus be honestly reflected by her CHC profile, which conveys information used by workers to detect high- and low-fecundity queens. Queens with low fecundity, a trait presumably communicated by having low levels of the di-methyl alkanes 5,11-diMeC_29_ and 5,11-diMeC_33_, might then be eliminated by workers, bringing an end to reproductive competition among nestmate queens. Such indirect effects on queen survival could evolve via selection acting on workers to maximise colony reproductive efficiency and could thus be evolutionarily stable.

In conclusion, our results support the idea that, in the Argentine ant, some form of reproductive inhibition exists among nestmate queens that leads to workers executing less productive queens, as suggested in previous research^26^. However, more studies are needed to assess the physiological and/or behavioural effects of the di-methyl alkanes identified as candidate queen pheromones to clarify their precise mode(s) of action and their links to differences in fecundity among nestmate Argentine ant queens. The results of this research should improve our understanding of how highly polygynous insect societies are regulated and maintained.

## Methods

### Colony collection and maintenance

In March 2016, we collected 32 queens from 8 Argentine ant colonies found on the southern edge of the Gavarres Massif, near the village of Santa Cristina d’Aro (NE Iberian Peninsula). Heller et al.^35^ found that, between October and June, a given Argentine ant nest may have additional nests within a radius of 3–4 m. Therefore, to ensure sample independence, we collected queens from colonies that were separated by at least 10 m. All the colonies belonged to the Main supercolony. We used the ants collected in the field to create 8 artificial colonies in the laboratory. These colonies were polygynous; they each contained 4 queens and approximately 1,200 workers (i.e., a ratio of 300 workers per queen) that all came from the same colony in the field. We used a ratio of 300 workers per queen because that is approximately the ratio that has been observed in nature in early spring (Keller et al.^36^). The queens were allowed to acclimate to polygynous conditions for one week before the experiment began. The artificial colonies were a variant of those described by Passera et al.^15^ and were kept in rectangular plastic boxes. The main box (180 × 115 × 35 mm) contained a layer of dry plaster of Paris and was laterally connected to a smaller box (75 × 50 × 25 mm) by a wick of cotton wool permanently in contact with a piece of cotton soaked in water. To prevent the ants from escaping, the inner walls of the main nest box were coated with liquid PTFE (Fluon). We fed the ants daily using a variant of the artificial diet described by Keller et al.^37^. More specifically, we replaced the hashed beef with royal jelly and the sugar with honey. The food was not coated with paraffin and was placed directly on the floor of the box. We knew this diet would be suitable for rearing Argentine ant colonies because it has been used successfully in previous studies (e.g., it results in healthy workers, healthy sexuals, and high queen fecundity)^25,26^. The relative humidity of the colonies was around 80%. The colonies were kept at 28 °C, the optimal temperature for queen oviposition in the Argentine ant^25^. The queens were marked using Uni Paint markers (Mitsubishi Pencil Co., LTD) so that they could be identified during the whole study.

### Do nestmate queens affect the reproductive contribution of individual queens?

To determine if the presence of several mated queens in the colony (i.e., polygynous conditions) affected the reproductive contributions of individual queens, we measured queen fecundity by quantifying oviposition rates and ovarian activity.

To verify that they were laying eggs, all the queens were isolated in a single test tube nest before the first measurement of oviposition rate. Following Abril et al.^13^, the tube nest consisted of a transparent plastic tube (70 mm in length × 10 mm in diameter) with a plastic lid. The inner side of the plastic lid was covered by a layer of dry plaster of Paris, which was connected by a wick of cotton wool to a small chamber filled with water. As a result, the inside of the tube remained permanently humid over the course of our observations. After 24 h had passed, we quantified oviposition rate: the number of eggs laid by each queen was counted using a binocular microscope. Then, over the course of two weeks, queens were isolated four times for a 24-h period to quantify their oviposition rates (i.e., the mean number of eggs laid by each queen based on four separate counts). After each isolation period, queens were returned to their artificial colonies.

Using the same method as Hannonen et al.^20^, we then classified each colony’s queens as having low or high fecundity based on their overall oviposition rates. If the four queens were equally responsible for egg-laying, we would expect each queen to contribute approximately 25% of the eggs laid. Thus, a queen was defined as having low fecundity if her reproductive contribution was less than 25% or as having high fecundity if her reproductive contribution was greater than 25%. Each queen was individually marked using Uni Paint marker pens (Mitsubishi Pencil Co., Ltd). The mark was placed on the dorsal surface of the abdomen, which allowed the queens to be easily identified throughout the study.

Six of the artificial colonies contained two high-fecundity queens and two low-fecundity queens; the other two groups had one high-fecundity queen and three low-fecundity queens and three high-fecundity queens and one low-fecundity queen, respectively (Fig. 1). Thus, overall, there were 18 high-fecundity queens and 18 low-fecundity queens. Three of the queens died after the fourth isolation period and were therefore not used in the subsequent analyses.

In the next phase of the experiment, seven high-fecundity queens and eight low-fecundity queens were randomly chosen for cuticular hydrocarbon (CHC) analysis (see below). The remaining queens (seven high-fecundity queens and seven low-fecundity queens) were then exposed to monogynous conditions (i.e. placed in a colony such that each focal queen was the only queen in that colony) to assess any changes in their oviposition rates. In total, we created 14 monogynous colonies: 7 containing the high-fecundity queens and 7 containing the low-fecundity queens. Each colony contained 300 workers, such that the queen-to-worker ratio was the same as in the polygynous colonies. All workers came from the same polygynous colony as the queen. The monogynous groups were allowed to acclimate for a week and were fed the same artificial diet as the polygynous groups. After the acclimation period, we measured each queen’s oviposition rate twice a week for two weeks.

Reproductive skew was estimated via the binomial B-index^38,39^. We calculated the index values from the total number of eggs laid by each queen over the course of the four trials. The calculations were carried out using Skew Calculator 2013^39^. With this index, positive values indicate skewed reproduction, negative values indicate shared reproduction, and values of zero indicate random reproductive skew^38^. We calculated B as well as the maximum (i.e., one queen monopolises reproduction) and minimum (i.e., all queens reproduce equally) possible values of B. As per Hammond et al.^40^, we also determined the values of the adjusted B-index (B_adj_) to control for colony-specific variation in the maximum and minimum values of B. B_adj_ was calculated by finding the absolute difference between the observed and minimum B values divided by the absolute difference between the maximum and minimum B values^40^.

We quantified the ovarian index (OI) values of high- and low-fecundity queens from polygynous and monogynous colonies using the length method described in Sledge et al.^41^ and Cini et al.^42^, which determines the relative mean length of the six longest oocytes in the ovaries of each individual. Queens were dissected in saline solution, and the measurements were performed using ImageJ software (NIH; http://rsb.info.nih.gov/ij).

### Do workers pay more attention to high-fecundity queens than low-fecundity queens?

During the polygynous phase of the experiment, we measured queen attractivity to workers. The four queens in each group were placed in a box that also contained 30 of their workers. The queens were separated from the workers by a mesh screen; while the workers could pass through the screen, the queens could not. Then, for an hour, the number of workers seen grooming, feeding, or antennating each queen was counted every five minutes. Using these data, mean worker attention was calculated using the equation from Hannonen et al.^20^:

$$\left[\sum\left({\text{w}}_{\rm i}/{\text{w}}_{{\rm tot}}\right)\right]/n$$

where w_i_ = the number of workers attending to the *i*_th_ queen, w_tot_ = the total number of workers attending to all the queens, and n = the number of observation periods (of which there were 12). We quantified the mean amount of worker attention given to each queen three times during the two-week experimental period, and we then calculated the mean of these three estimates.

### Do high- and low-fecundity queens have different cuticular hydrocarbon profiles under polygynous versus monogynous conditions?

As described above, we randomly selected seven high-fecundity queens and eight low-fecundity queens after the polygynous phase of the experiment for CHC profiling. We also characterised the CHC profiles of the seven high-fecundity queens and seven low-fecundity queens that were used in the monogynous phase of the experiment. To characterize the CHC profiles we used the same methodology as in Abril and Gómez^26^. In brief, all the samples were frozen (− 20 °C) until CHC extraction could occur. The queens’ gasters were removed to prevent any contamination by the Dufour gland and were stored separately until we could quantify OI values. Consequently, we extracted CHCs from samples comprising the queens’ heads, thoraces, and legs. First, we immersed the samples in 50 μl of dichloromethane (GC grade) for 10 min. The queens’ bodies were then removed, and the extract was stored at − 20 °C until further analysis could take place. The extracts were evaporated and then redissolved in 50 μl of dichloromethane containing eicosane (8 ng/μl) as an internal standard. Next, 1 μl of each extract was injected into a gas chromatography (GC; Agilent 7820A Series, Agilent Technologies, USA) equipped with a HP-5 capillary column (30 m × 0.32 mm × 0.25 µm) and a flame ionisation detector. Sample analysis was performed using helium as a carrier gas; the flow rate was set to 2 ml/min (34.9 cm/s); an injection volume of 1 µl with a split ratio of 1:5 was used; and the inlet temperature was set to 275 °C. The following temperature program was employed: 75 °C held for 0 min; an increase from 75 to 200 °C at a rate of 20 °C min^−1^; then another increase from 200 to 315 °C at a rate of 5 °C min^−1^; and 315 °C held for 15 min. The FID detector was set to a temperature of 300 °C. CHCs were identified using standard alkanes, compound libraries, and Kovats retention indices. CHC quantities were determined using the area of eicosane, which was the internal standard (ng per head and thorax [i.e., one queen]).

### Statistical analyses

R^43^, Past^44^, and Statistica^45^ were used to perform all the statistical analyses and create all the figures.

Generalised linear mixed models (GLMMs) were used to compare the oviposition rates (Poisson error distribution and log-link function using the MASS package and the glmmPQL function) and the OI values (Gaussian error distribution and identity link function using the nlme package and lme function) of high- and low-fecundity queens under polygynous versus monogynous conditions. We included colony as a random factor. Two separate analyses were run to compare oviposition rates and OI values between the high- and low-fecundity queens under the two sets of conditions.

Worker attention paid to high- versus low-fecundity queens under polygynous conditions was also compared using GLMMs (Gaussian error distribution and identity link function using the nlme package and lme function). The response variable was worker attention; queen type (i.e., high or low fecundity) was a fixed factor, and colony identity was a random factor.

As in Abril and Gómez^26^, to characterise the queens’ CHC profiles, we determined the relative contribution of each CHC peak. Only identified peaks whose mean relative quantities were above 1% in at least one of the groups of queens were used. Discriminant analyses were then performed to investigate how CHC profiles differed between high- and low-fecundity queens under polygynous and monogynous conditions. To assess differences among the four groups of queens, we performed an analysis of similarity (ANOSIM) followed by pairwise tests. To identify the CHCs that characterised each group, principal component analysis (PCA) was used. We focused on the CHCs demonstrating the greatest differences—those whose factor loadings on the first axis had an absolute value > 0.80. We further explored these data using GLMMs (Gaussian error distribution and identity link function using the nlme package and lme function) in which the response variable was the mass of a given peak in nanograms (calculated by comparing the area of the peak with that of the internal standard) and colony identity was a random factor. Finally, we examined correlations between the absolute quantities (ng) of the different CHCs and both oviposition rates and OI values using Pearson correlation tests.

We used Cohen's d as a measure of effect size in all mean comparisons. Cohen^46^ proposed a scale to assess the magnitude of the effect being: *d* < 0.2 negligible, *d* < 0.5 small, *d* < 0.8 medium and *d* > 0.8 large.

To ensure normality, all the non-normally distributed variables were log-transformed prior to being used in the discriminant analyses and the PCA. In addition, Levene’s test was used to determine whether the variables displayed homogeneity of variance; only those that did were retained.

## Data Availability

The datasets generated and analysed during the current study are available from the corresponding author upon request.
